# Epidemiological evidence on extra-medical use of prescription pain relievers: transitions from newly incident use to dependence among 12–21 year olds in the United States using meta-analysis, 2002–13

**DOI:** 10.7717/peerj.1340

**Published:** 2015-10-20

**Authors:** Maria A. Parker, James C. Anthony

**Affiliations:** Department of Epidemiology & Biostatistics, Michigan State University, East Lansing, MI, United States

**Keywords:** Prescription pain relievers, Opioids, Adolescents, Dependence, Transition, Epidemiology, Incidence

## Abstract

**Background.** When 12-to-21-year-olds start using prescription pain relievers extra-medically, some of them transition into opioid dependence within 12 months after such use. Our main aim for this epidemiological research on 12-to-21-year-olds in the United States (US) is to estimate the risk of becoming a newly incident case of opioid dependence within 12 months after onset of using prescription pain relievers extra-medically (EMPPR).

**Methods.** Meta-analyses from multiple independent replication samples now are possible, based upon nationally representative survey samples of US adolescents age 12–21 years. All 12-to-21-year-olds were sampled and recruited for the US National Surveys on Drug Use and Health, with standardized assessments of EMPPR use and opioid dependence (NSDUH, 2002–2013).

**Results.** Peak risk for a transition from start of EMPPR use to opioid dependence within 12 months is seen at mid-adolescence among 14-to-15-year-olds (6.3%, 8.7% per year), somewhat earlier than peak risk for starting EMPPR use (seen for 16-to-19-year-olds at 4.1%, 5.9% per year). Applied to 12-to-21-year-olds in the US between 2002–2013, an estimated 8 million started using PPR extra-medically. Each year, roughly 42,000 to 58,000 transitioned into opioid dependence within 12 months after onset of such use.

**Discussion.** These epidemiological estimates for the US in recent years teach us to expect one transition into adolescent-onset opioid dependence within 12 months for every 11–16 newly incident EMPPR users, yielding perhaps 120 newly incident opioid dependent cases in need of practitioner attention or treatment services, each day of each year. This evidence can be used to motivate more effective public health prevention, outreach, and early intervention programs as might prevent or delay occurrence of EMPPR use and opioid dependence.

## Background

The United States has seen dramatic growth in numbers of overdoses and overdose deaths attributed to prescription pain relievers (PPR), a drug subtype that consists mainly of prescription-type opioid drug compounds with trade names such as Vicodin^®^, Lortab^®^, Lorcet^®^, Darvocet^®^, Darvon^®^, Tylenol^®^ with Codeine, Percocet^®^, Percodan^®^, Tylox^®^, and OxyContin^®^ (United States Centers for Disease Control and Prevention, 2014). Supplemental Information 1 provides a more comprehensive list of the compounds termed ‘prescription pain relievers’ in research of this type.

All too often, these overdoses are occurring after 12-to-21-year-olds have started to use PPR extra-medically (i.e., to get high and for related feelings and experiences, or otherwise outside the boundaries of a prescriber’s intent, as defined in Anthony, Warner & Kessler, 1994). Hereinafter, PPR is the acronym for ‘prescription pain reliever’ and ‘extra-medical prescription pain reliever’ is designated by the abbreviation EMPPR. Sidebar 1 and Supplemental Information 2 provide operational specifications for the more general concept of extra-medical drug use.

For the most part, growth in numbers of PPR overdoses in the United States (US) can be traced back to extra-medical use of these compounds as opposed to taking medicines exactly as prescribed (Volkow et al., 2011; Manchikanti et al., 2012; United States Department of Health and Human Services, 2014; United States Centers for Disease Control and Prevention, 2014; King et al., 2014). In recent years, more than four percent of 12-to-21-year-olds qualify as recently active EMPPR users (United States Department of Health and Human Services, 2014; United States Department of Health and Human Services, 2015). As gauged by expected values based on US experiences between 1980 and 1999, it now is legitimate to speak of a 21st century ‘epidemic’ of EMPPR use among young people in this country.

Underlying fundamental conditions and processes giving rise to this epidemic are being investigated, and new interventions to prevent EMPPR use and to reduce diversion of legitimately prescribed PPR have been developed (Compton & Volkow, 2006; Cicero & Ellis, 2015; Dart et al., 2015; Kanouse & Compton, 2015; Maxwell, 2015). In addition, once EMPPR use occurs, there is a risk that a syndrome in the form of opioid dependence will occur, as noted elsewhere (Anthony, Warner & Kessler, 1994; Martins, Ghandour & Chilcoat, 2007). In most research projects on this topic, the concept of opioid dependence has been specified in relation to the Fourth Edition of the American Psychiatric Association’s Diagnostic and Statistical Manual, American Psychiatric Association, 1994; DSM-IV).

One recent contribution to epidemiological evidence about EMPPR use in the United States was made by Meier and colleagues (2012), who discovered a peak risk of becoming a newly incident EMPPR user during the adolescent years from age 12 to 21. Those estimates were based on data gathered from 2004 to 2008, using what Seedall & Anthony (2015) have called a ‘mutoscope’ approach that can be used to trace the experience of each adolescent birth cohort forward, year by year (Meier, Troost & Anthony, 2012). For 12-to-21-year-olds, results for each birth cohort showed peak values of risk at mid-adolescence (roughly age 16 years), followed by declining incidence rates across the later adolescent years (Meier, Troost & Anthony, 2012).

In this new report on the epidemiology of EMPPR use, also with a focus on 12-to-21-year-olds in the United States, the primary aim is to estimate risk of becoming a newly incident case of DSM-IV-type opioid dependence not too long after EMPPR use starts (i.e., within 12 months). We also update EMPPR risk estimates, extending through 2013 the prior 2004–2008 estimates of Meier, Troost & Anthony (2012).

The value of epidemiological estimates of this type can be seen in their utility as motivators for new or renewed efforts to prevent and control the occurrence of EMPPR use. Estimates of this type also can be used to motivate early outreach and intervention efforts that are needed to identify and help the new EMPPR users who develop opioid dependence (Compton & Volkow, 2006).

Sidebar 1 ‘Extra-Medical’ or ‘Extramedical’ Drug Use in Drug Dependence Epidemiology

The concepts of ‘extra-medical’ (or ‘extramedical’) drug use were introduced in 1989 by a Johns Hopkins University research work group supported by the United States National Institute on Drug Abuse and led by James C. Anthony, then Professor with appointments in the Johns Hopkins University School of Hygiene and Public Health (Mental Hygiene; Epidemiology) and its School of Medicine (Psychiatry and Behavioral Sciences). The research group provided a set of operational specifications for epidemiological field surveys, and proposed a set of pre-written standardized survey items for use in the first United States National Comorbidity Survey (NCS-1), scheduled for completion in 1990–92.

By creating a new term, the research group was trying to avoid ambiguities and other problems of signification encountered when terms such as ‘drug misuse’ and ‘non-medical drug use’ and ‘drug abuse’ appeared in the scientific literature. The group thought that these ambiguities and signification problems might be avoided by introducing a completely new term with clear operational specifications.

The team offered this introduction, to be presented to survey participants before its set of proposed standardized survey items on extra-medical drug use:

We are interested in the extra-medical use of these prescription-type drugs.

Extra-medical use is any use on your own; that is, either:

**One**, without a doctor’s prescription, or

**Two**, in greater amounts than prescribed, or

**Three**, more often than prescribed, or

**Four**, for any reasons other than a doctor said you should take them—such as for kicks, to get high, to feel good, or curiosity about the pill’s effect.

The term ‘extramedical drug use’ first appeared in the peer reviewed scientific literature during 1994, within a journal article on drug dependence epidemiology (Anthony, Warner & Kessler, 1994 ^*^). This article has been cited more than 1,000 times, and at present is cited at a rate of 35–50 citations per year (according to Google Scholar). The term has been adopted by multiple US and international research groups.

***Anthony JC, Warner LA, Kessler RC. 1994.** Comparative epidemiology of dependence on tobacco, alcohol, controlled substances, and inhalants: basic findings from the national comorbidity survey. *Experimental and Clinical Psychopharmacology* **2(3)**: 244–268.A footnote of interest: When the NCS-1 interview schedule was printed, an unnamed member of the NCS-1 field research team decided that ‘non-medical’ use was intended rather than ‘extra-medical’ use. As a result, in the NCS-1 codebook, the group’s suggested introduction appears with ‘non-medical’ substituted for ‘extra-medical’: (http://www.icpsr.umich.edu/cgi-bin/file?comp=none&study=6693&ds=1&file_id=946014&path=NAHDAP), last accessed 8 September 2015.

## Materials & Methods

The study population for this investigation consists of 12-to-21-year-old noninstitutionalized civilian residents in all 50 states and the District of Columbia during the early 21st century, as sampled and surveyed each year by research teams supported by the US Substance Abuse and Mental Health Services Administration (SAMHSA) during 2002–2013. Each year’s community probability sample consisted of survey participants age 12 years and older, after regularized sampling, recruitment, informed consent/assent, and assessment protocols that followed a human subjects protection approach approved by cognizant Institutional Review Boards, as already described in multiple prior reports (e.g., Meier, Troost & Anthony, 2012; United States Department of Health and Human Services, 2014; United States Department of Health and Human Services, 2015; Vsevolozhskaya & Anthony, 2014). The NSDUH sampling frame is noteworthy because it encompasses noninstitutionalized adolescents irrespective of school attendance and wherever they are living, not only in households but also in non-institutional group quarters, dwelling units such as homeless shelters, and college dormitories.

Unweighted numbers of designated 12–21 year old participants in each year’s multi-stage area probability sample range from 41,248 to 42,864 (United States Department of Health and Human Services, 2013). Survey participation levels for this age group are ∼75% (United States Department of Health and Human Services, 2015). Fig. S1 provides more details on approximate unweighted sample sizes.

The NSDUH field survey assessment has made use of audio computer assisted self-interview methods (ACASI), with multiple modules across a range of drug use and health topics, and with coverage of prescription pain relievers via PPR module items listed in Supplemental Information 1. PPR module items have been used to identify newly incident EMPPR users as well as those who had become opioid dependence cases within 12 months after onset of EMPPR use (Martins, Ghandour & Chilcoat, 2007; Meier, Troost & Anthony, 2012; United States Department of Health and Human Services, 2015). A generally supportive series of diagnostic reliability and validity estimates for this type of assessment has been reported by independent research teams (Anthony et al., 1985; Grant et al., 1995; Compton et al., 2013).

Whereas NSDUH assessments do not ask about lifetime history of opioid dependence, the survey items have identified newly incident EMPPR users who do and do not qualify as cases of opioid dependence as observed to be present within 12 months after onset of EMPPR use. Based on these assessments, it is possible to identify the newly incident EMPPR users (i.e., those who started EMPPR use within the 12 months prior to assessment), and to measure opioid dependence that occurs after onset of EMPPR use (i.e., within the same 12 month interval).

The analysis plan started with Tukey-style exploratory data analysis steps and inspection of analysis-weighted marginal distributions. Thereafter, analysis-weighted estimates of annual incidence rates for EMPPR use were derived, followed by estimation of transition probabilities (with standard errors) for how often opioid dependence cases were observed after onset of EMPPR use (i.e., within 12 months).

Our initial attempt to produce year-by-year estimates was thwarted by the small number of opioid dependence cases observed each year, with resulting unstable variance estimates. For this reason, we created year-pairs as well as corresponding age-pairs (Parker & Anthony, 2014). This approach produced acceptable stability in the variance estimates. Resulting year-pair estimates for incidence proportions and for the variance of the incidence proportions are appropriate for meta-analysis because each year’s sample can serve as a new and statistically independent replication. Accordingly, for meta-analysis purposes, we treated each age-pair and year-pair as an independent replication sample and source of meta-analysis data, building from prior work by Meier, Troost & Anthony (2012) and DeAndrea, Troost & Anthony (2013).

Except as noted, the meta-analysis confidence intervals are from ‘fixed effects’ estimators. When the heterogeneity test statistic suggested potentially important variations (i.e., *p* < 0.05), the ‘random effects’ estimator also is shown. All estimates are analysis-weighted with Taylor series linearization for variance estimation. Meta-analyses are based on Stata Version 13 ‘metan’ commands (StataCorp, 2013), with a logit transformation suggested by Vsevolozhskaya & Anthony (2014).

These study estimates might be of special interest to practitioners interested in prevention of opioid dependence, but of course constraints on generalizability deserve mention. Our discussion addresses issues of generalizability, and whether this study’s estimates for the nation as a whole might be useful in the context of the work of officials responsible for individual public health districts and states, given what is known about observed state-level variations in the incidence of EMPPR use (Vsevolozhskaya & Anthony, 2014).

## Results

Table 1(A) describes the sample of 12-to-21-year-olds. It cross-tabulates effective sample sizes to illustrate unweighted numbers of newly incident EMPPR users in the sample, disclosing peak values between age 14 and age 17 years. Essentially the same peaks are seen in the weighted counts of Table 1(B) and in the analysis-weighted estimates of Table 2.

**Table 1:** Approximate unweighted numbers of newly incident adolescent onset extra-medical users of prescription pain relievers per subgroup (A) and weighted population counts (B) for newly incident extra-medical prescription pain reliever users by age and year-pair. Data from Restricted-use Data Analysis System subsamples of the National Surveys on Drug Use and Health, United States 2002–2013.

Year pair	12–13 y	14–15 y	16–17 y	18–19 y	20–21 y
**(A) Approximate number of newly incident users in the sample**					
2002–2003	191	625	814	564	308
2004–2005	199	520	732	477	303
2006–2007	171	476	681	532	263
2008–2009	145	546	708	514	285
2010–2011	157	446	679	426	249
2012–2013	118	353	507	313	260
**(B) Corresponding weighted count of newly incident users in the US population**					
2002–2003	104,000	344,000	454,000	397,000	222,000
2004–2005	113,000	301,000	430,000	351,000	224,000
2006–2007	98,000	276,000	397,000	418,000	205,000
2008–2009	81,000	313,000	408,000	392,000	215,000
2010–2011	86,000	248,000	378,000	329,000	196,000
2012–2013	66,000	202,000	290,000	254,000	208,000
Estimated analysis-weighted total (per 100)	548,000	1,684,000	2,357,000	2,141,000	1,270,000

**Table 2:** Estimated risk of becoming a newly incident extra-medical user of prescription pain relievers, stratified by age at assessment and survey year-pair. Age- and time-specific incidence estimates (A), 95% confidence intervals (B), and age-specific meta-analysis summary estimates. Data from Restricted-Use Data Analysis System subsamples of the National Surveys on Drug Use and Health, United States 2002–2013.

Year pair	12–13 y	14–15 y	16–17 y	18–19 y	20–21 y
**(A) Estimated risk of becoming a newly incident user (per 100)**					
2002–2003	1.3	4.4	6.3	5.7	3.5
2004–2005	1.4	3.7	5.9	4.9	3.7
2006–2007	1.2	3.4	5.2	5.6	3.3
2008–2009	1.1	3.9	5.3	5.0	3.4
2010–2011	1.1	3.2	4.9	4.3	2.8
2012–2013	0.8	2.5	3.8	3.3	3.0
**(B) 95% confidence intervals for estimates in (A) (per 100)**					
2002–2003	1.1, 1.5	4.1, 4.8	5.9, 6.8	5.2, 6.2	3.1, 3.9
2004–2005	1.2, 1.6	3.4, 4.0	5.5, 6.3	4.5, 5.4	3.3, 4.1
2006–2007	1.1, 1.4	3.1, 3.7	4.9, 5.6	5.2, 6.1	2.9, 3.7
2008–2009	0.9, 1.3	3.6, 4.2	4.9, 5.7	4.6, 5.4	3.1, 3.9
2010–2011	1.0, 1.3	2.9, 3.5	4.6, 5.3	3.9, 4.7	2.5, 3.2
2012–2013	0.7, 1.1	2.2, 2.9	3.4, 4.2	2.9, 3.8	2.5, 3.5
Meta-analysis summary estimates & 95% confidence intervals (per 100)^a^	1.2 (1.0, 1.3)	3.5 (3.0, 4.0)	5.2 (4.5, 5.9)	4.8 (4.1, 5.5)	3.3 (3.1, 3.5)^b^

**Notes.**

a Supplemental Information 4 provides additional information about I-squared.

b Here, the I-squared statistic has 0.05 > *p* > 0.15 so the 95% CI are from ‘fixed effects’ estimation; the corresponding ‘Random Effects’ interval is 3.0, 3.6. All other meta-analytic 95% CI are from ‘random effects’ estimation (due to I-squared *p* < 0.05).

Diagonal cells of these tables also provide what has been called an ‘epidemiological mutoscope’ view of the experience of individual cohorts. To illustrate, in 2002–3, an estimated 1.1-to-1.5 percent of 12-to-13-year-olds had just started EMPPR use. Followed forward to its 2004–5 completely independent re-sample, that same cohort had turned 14–15 years old, and cohort-specific risk of EMPPR use had increased to 3.4-to-4.0%. Then, with a new re-sample, and observed at age 16–17 years in 2006–7, estimated incidence of EMPPR use for the same cohort is 4.9-to-5.6%, not appreciably distant from the 4.6-to-5.4% estimates observed in 2008–9 when the cohort had turned age 18–19 years old. Thereafter, the cohort-specific risk of becoming an EMPPR user dropped to the 2.5-to-3.2% level in 2010–11. Followed down its diagonal in Table 2, the cohort-specific pattern for 12–13 year olds in 2004–5 is not appreciably different from what can be seen for 12–13 year olds observed in 2002–3. (Seedall & Anthony (2015) provide additional details about this epidemiological mutoscope view of each cohort, which complements what can be learned by studying the row and column totals of each table of this type.)

With evidence borrowed from all years, the age-specific meta-analysis summary estimates presented in Table 2 (bottom row) make it clear that no more than about one percent of 12–13 year olds became newly incident EMPPR users in these years. The meta-analysis summary estimates disclosed a substantial upward jump in incidence from age 12–13 years to age 14–15 years, followed by another substantial jump to peak point estimates at age 16–17 years and age 18–19 years, followed by a statistically robust decline in risk for the 20-to-21-year-olds.

Table 2 also might be disclosing a secular trend that merits continuing attention in future years. The peak values for newly incident EMPPR use among 16-to-17-year-olds in 2012–13 are tangibly smaller than corresponding values for prior years, as gauged by non-overlap of confidence intervals (CI). Forest plots presented for age-groups in Fig. S2 lead to a similar conclusion.

Table 3 shifts attention to risk of becoming opioid dependent within 12 months after onset of EMPPR use. The estimated risk estimates for the age-pair at 14-to-15-years-old is noteworthy in three ways. First, viewed row-wise, except for 2010–11, every year-pair shown in Table 3(A) discloses a peak point estimate for becoming a case of opioid dependence at age 14–15 years, Second, meta-analysis summary estimates for the 14-to-15-year-old newly incident EMPPR users are robustly larger than estimates observed for the other age-pairs. Third, with no more than one exception, the epidemiological mutoscope view of the table’s diagonal cells shows that peak incidence of opioid dependence for birth cohorts (given newly incident EMPPR use) is larger at age 14–15 years than at other ages.

**Table 3:** Estimated risk of transitioning and becoming an opioid dependence case no longer than 12 months after onset of starting to use prescription pain relievers extra-medically. Estimated risk of transitioning to opioid dependence (A), 95% confidence intervals (B), and age-specific meta-analysis summary estimates. Data from Restricted-use Data Analysis System subsamples of the National Surveys on Drug Use and Heath, United States 2002–2013.

Year pair	12–13 y	14–15 y	16–17 y	18–19 y	20–21 y
**(A) Estimated risk of becoming an opioid dependence case (per 100 newly incident EMPPR users)**					
2002–2003	4.2	6.3	4.7	2.8	1.8
2004–2005	3.2	9.8	5.1	3.3	2.4
2006–2007	5.2	7.9	3.9	1.7	3.4
2008–2009	5.4	7.5	4.8	4.2	3.2
2010–2011	8.2	5.8	7.1	6.1	2.4
2012–2013	5.1	6.4	6.1	4.8	2.1
**(B) 95% confidence intervals for (A) estimates (per 100)**					
2002–2003	2.3, 7.4	4.3, 9.1	3.1, 7.1	1.5, 5.1	0.9, 3.7
2004–2005	1.7, 6.0	6.9, 13.6	3.4, 7.6	1.8, 5.9	1.1, 5.0
2006–2007	2.8, 9.7	5.2, 11.7	2.3, 6.5	0.8, 3.4	1.5, 7.7
2008–2009	2.6, 11.0	5.4, 10.4	3.4, 6.7	2.4, 7.3	1.3, 7.8
2010–2011	4.4, 14.7	3.6, 9.2	4.7, 10.7	3.4, 10.7	0.9, 6.1
2012–2013	2.6, 15.1	3.8, 10.4	3.9, 9.5	2.4, 9.2	0.8, 5.2
Meta-analysis summary estimates & 95% confidence intervals (per 100)^a^	5.1 (3.9, 6.6)	7.4 (6.3, 8.7)	5.2 (4.4, 6.2)	3.6 (2.5, 5.0)^b^	2.4 (1.7, 3.4)

**Notes.**

a Supplemental Information 4 provides additional information about I-squared.

b Here, the I-squared statistic has 0.05 < *p* < 0.15 so the 95% CI are from ‘random effects’ estimation; the corresponding ’fixed effects’ interval is 2.8, 4.6. All other meta-analytic 95% CI are from ‘fixed effects’ estimation (due to I-squared *p* > 0.15).

The epidemiological estimates presented in Tables 2 and 3 seem to be quite clear in their implications for age-specific timing of public health interventions. Delay of outreach programs and interventions until the adult years, with failure to concentrate resources on teenagers, might well be misguided, if these incidence estimates are judged to be trustworthy and actionable.

For a variety of reasons, including the technical detail known as right-censoring in the context of survival analyses, it can be difficult to estimate the mean duration of EMPPR use. By combining this study’s annual incidence estimates with separately published prevalence estimates (United States Department of Health and Human Services, 2006)–(United States Department of Health and Human Services, 2015), it is possible to derive such estimates for EMPPR use via a functional relationship that expresses prevalence as a function of incidence times mean duration. Formulated in this fashion, when EMPPR use starts during the adolescent years, its mean duration can be estimated as 2–4 years. Tables S1 and S2 provide details about these calculations.

## Discussion & Conclusions

For scientists who study prescription pain relievers, policy-makers, regulatory bodies and health professionals, this report’s characterization of a 21st century ‘epidemic’ may be useful. Consistent with prior estimates through 2008 (e.g., Meier, Troost & Anthony, 2012), years of peak risk for starting extra-medical PPR use are observed during mid-adolescence.

When this study’s epidemiological estimates are applied to population counts for 12–21 year olds in the US, the calculations suggest that roughly 8 million adolescents started using PPR extra-medically between 2002 and 2013. In addition, during each year between 2002 and 2013, roughly 42,000 to 58,000 became newly incident cases of opioid dependence within 12 months after onset of extra-medical use (i.e., at least 120 cases per day). One can presume that all or most of these cases might be in need of advice or monitoring by a general practitioner, if not more intensive drug treatment services.

At 2.4% (95% CI [1.7%, 3.4%]), the estimated risk for becoming an opioid dependence case within 12 months after onset of EMPPR use is relatively low among 20-to-21-year-olds. Opioid dependence risk estimates seen for newly incident 12-to-17-year-old EMPPR users are 2–3 times larger, possibly reflecting a more general susceptibility to complications when drug use starts early, as seen elsewhere (e.g., Anthony & Petronis, 1995). An apparent peak risk for transition to opioid dependence within 12 months at age 14–15 years is noteworthy. In the US, by age 14–15 years, most teenagers have qualified for admission to the secondary school level. Perhaps PPR become more readily available once primary school years have passed.

In this report, by joining previously published age-specific estimates for prevalence of EMPPR use with this study’s newly published age-specific estimates of incidence rates through 2013, it has been possible to discover that for the most part the mean duration of extra-medical PPR use is on the order of 2–4 years, although an allowance for age-related variation must be made. Previously published estimates suggest that many EMPPR users try these compounds no more than a few times and then stop, with duration far shorter than the estimated mean, whereas others become persistent users, with duration considerably longer than the estimated mean (e.g., those who become opioid dependence cases). We regret that statistically reliable age-specific estimates for the duration of opioid dependence attributable to PPR cannot be derived from these NSDUH data.

Public health researchers, as well as practitioners interested in prevention and control of PPR use and dependence, can use this study’s estimates in attempts to marshal new resources for clinical and population health initiatives. Estimates of this type might help motivate design, implementation, and evaluation of more effective public health outreach and early intervention services for adolescent-onset users and opioid dependence cases in the community.

Among study limitations, we note the self-report character of NSDUH data, for which we have no logistically feasible alternative in nationally representative community sample surveys. While it is true that ACASI assessment protocols qualify as ‘best practices’ for large sample quantitative survey research on generally illegal and sensitive behaviors, some young people in these samples might fail to disclose newly incident EMPPR use or clinical features of opioid dependence once it occurs (i.e., lapses in field assessment ‘sensitivity’). In addition, not all EMPPR users are assessed exactly one year after onset of EMPPR use. As such, this study’s estimates fall somewhat short of ‘annual’ incidence rates. R-DAS estimation does not make it possible to calculate person-months from first day of EMPPR use to NSDUH assessment dates (United States Department of Health and Human Services, 2014). In addition, the technical detail of post-stratification adjustment to US Census cell counts is allowed to vary across R-DAS year pairs. In consequence, comparisons across NSDUH year-pairs might show variations due to variations in the US Census cell counts. As such, some degree of caution is needed when comparing R-DAS estimates across year-pairs.

With respect to the measurement issues just noted, a lack of specificity might counter-balance departures from 100% sensitivity. Some adolescents might exaggerate and boast about EMPPR experiences that never truly occurred. Alternately, some might misunderstand survey questions in a direction that creates specificity errors. Therefore, as is true for almost all epidemiological surveillance estimates, the large-sample scale for survey coverage required to achieve nationally representative probability samples tends to thwart deep probes into qualitative research issues of screening or diagnostic validity as might be achieved via drug toxicology assays or a standardized clinical reappraisal work-up of a type made feasible in research with smaller and more local samples. Nonetheless, in more than 30 years of standardized clinical reappraisal research on large sample survey-based diagnostic assessments of DSM-type drug dependence, validity evidence has generally been supportive, often more supportive than has been true for other DSM categories of neuropsychiatric disorders, as noted in articles cited within our Methods section.

In any community sample survey of drug outcomes, there is a possibility of omissions of seriously disabled opioid-dependent users with lives so disrupted that they qualify as non-participants in the survey operations, even when their names are included on community survey sampling rosters. This is a methodological issue that pertains to the sensitivity of the surveillance operation, and is not an issue of the sensitivity of the survey measurements *per se*. To the extent that this research project involves estimation of the probability of transitioning from first onset of use into opioid dependence, there might a slight under-estimation of these probabilities, due to left-censoring of severely affected cases of this type. A topic of continuing investigation, not yet resolved, is how often users move quickly from first use into dependence syndromes that are disabling to the point of survey non-participation. Our expectation is for a small downward bias in the estimates, given that they generally are based upon users in the earliest months of the opioid dependence process. A related omission involves fatal overdose deaths as might occur on the first or second use of the drug, in which case these users can be missing from the survey denominators altogether. US vital statistics, to date, suggest that no more than a handful of such deaths would be missed by NSDUH field survey operations, given that the number of prescription opioid overdose deaths in the US totals no more than about 16,000 for the country as a whole, with a total population size of roughly 320 million individuals (United States Centers for Disease Control and Prevention, 2014). A recent study of the potential ‘Len Bias’ bias suggests negligible error from this theoretically interesting source of epidemiological bias (Lopez-Quintero et al., 2015).

Readers interested in dependence that occurs after strictly medical use of PPR may be disappointed that NSDUH focuses exclusively on extra-medical PPR use. One must look elsewhere for opioid dependence estimates when users stay well within boundaries of a prescribing clinician’s instructions. It seems reasonable that this study’s estimates of opioid dependence transition probabilities might be larger than what would be observed in research on PPR use exactly as prescribed, because the susceptibility traits giving rise to adolescent-onset extra-medical drug use almost certainly overlap with those influencing adolescent-onset drug dependence. Nonetheless, there now are no definitive nation-level estimates for these transition probabilities in the context of medically prescribed use. Estimates of this type will be needed to make a direct comparison of dependence risks across these different contexts of medical and EMPPR use.

We acknowledge that the relationship between PPR use and opioid dependence can be complex, with feedback loops. Quite clearly, EM use can lead to dependence, but dependence that develops during medically prescribed use also can lead to EMPPR use, as discussed elsewhere (e.g., Anthony, 2010).

Another question is whether these estimates for the US as a whole might generalize to sub-units as small as public health districts, to jurisdictions as large as states or regions within the US, or to other countries. There is substantial evidence of state-level variations in the occurrence of EMPPR use (Vsevolozhskaya & Anthony, 2014). Observed variations in those estimates lead us to hesitate before recommending application of this study’s estimates to state or sub-state units. Fortunately, R-DAS 10-year datasets (2002–2011) make provisions for sub-state estimates. For the larger sub-states (and for states), it is possible to pool data across 10 years in order to derive summary estimates. As for applicability elsewhere (i.e., in other countries), we urge caution.

Notwithstanding limitations of this type, and the fact that the transition probabilities for onset of opioid dependence within 12 months actually might be somewhat larger than we have estimated, the epidemiological estimates observed here are not trivial. These estimates deserve both clinical and public health attention. These estimates should help encourage and motivate the US National Institute on Drug Abuse, Centers for Disease Prevention and Control, and others to focus or renew attention to 12–21 year olds in current efforts to prevent and control extra-medical use of prescription pain relievers and associated opioid dependence risks.

## Supplemental Information

10.7717/peerj.1340/supp-1 Supplemental Information 1 National Surveys on Drug Use and Health (NSDUH) Assessments

10.7717/peerj.1340/supp-2 Supplemental Information 2 Background and specifications of ‘extra-medical’ drug use

10.7717/peerj.1340/supp-3 Figure S1 Flow chart diagram of 12–21 year old newly incident extra-medical prescription pain relievers users and those who transition to dependence within 12 months Data from the Survey Documentation and Analysis System of the National Surveys on Drug Use and Heath, United States 2002–2013. The NSDUH Restricted-use Data Analysis System (R-DAS) provides only weighted population estimates, but approximate unweighted sample sizes can be derived from NSDUH public use datasets archived with the Inter-university Consortium for Political and Social Research, as shown in this flow diagram.

10.7717/peerj.1340/supp-4 Supplemental Information 4 Meta-analysis specifications for ‘metan’ command

10.7717/peerj.1340/supp-5 Figure S2 Age-specific meta-analysis forest plots for the estimated risk of becoming a newly incident extra-medical prescription pain reliever user and making a transition to opioid dependence within 12 months The estimated risk of becoming a newly incident extra-medical user of prescription pain reliever drugs (A, leftmost columns) and the estimated risk of making a rapid transition and becoming an opioid dependence case no longer than 12 months after onset of starting to use these opioids extra-medically (B, rightmost columns). Risk estimates & 95% confidence intervals (per 100). Data from Restricted-use Data Analysis System subsamples of the National Surveys on Drug Use and Heath, United States 2002–2013.

10.7717/peerj.1340/supp-6 Table S1 Estimated prevalence of being an extra-medical user of prescription pain relievers during the year prior to assessment, stratified by age at assessment and survey year pair Age- and time-specific incidence estimates (A), 95% confidence intervals (B), and age-specific meta-analysis summary estimates. Data from Restricted-use Data Analysis System subsamples of the National Surveys on Drug Use and Heath, United States 2002–2013.

10.7717/peerj.1340/supp-7 Table S2 Mean duration of extra-medical prescription pain reliever use by age Data from Restricted-use Data Analysis System subsamples of the National Surveys on Drug Use and Heath, United States 2002–2013. Offer Letter *Estimate derived via division of the Prevalence Estimate by the Incidence Estimate.

## Additional Information and Declarations

### Data Availability

The following information was supplied regarding data availability: Release of original raw data creates a ‘disclosure’ potential such that values of 3 + variables might be combined in order to identify a specific individual participant. Interested scientists can request the R-DAS analysis results in spreadsheet form by emailing mparker@epi.msu.edu. The spreadsheet cells contain values for the multi-way cells from the cross-tabulations produced by R-DAS, upon which our study estimates are based.

## References

[ref-1] American Psychiatric Association (1994). DSM-IV: Diagnostic and Statistical Manual of Mental Disorders.

[ref-2] Anthony JC (2010). Novel phenotype issues raised in cross-national epidemiological research on drug dependence. Annals of the New York Academy of Sciences.

[ref-3] Anthony JC, Folstein M, Romanoski AJ, Von Korff MR, Nestadt GR, Chahal R, Merchant A, Brown CH, Shapiro S, Kramer M, Gruenberg EM (1985). Comparison of the lay diagnostic interview schedule and a standardized psychiatric diagnosis. Experience in eastern Baltimore. Archives of General Psychiatry.

[ref-4] Anthony JC, Petronis KR (1995). Early-onset drug use and risk of later drug problems. Drug and Alcohol Dependence.

[ref-5] Anthony JC, Warner LA, Kessler RC (1994). Comparative epidemiology of dependence on tobacco, alcohol, controlled substances, and inhalants: basic findings from the National Comorbidity Survey. Experimental and Clinical Psychopharmacology.

[ref-6] Cicero TJ, Ellis MS (2015). Abuse-deterrent formulations and the prescription opioid abuse epidemic in the United States: lessons learned from oxycontin. JAMA Psychiatry.

[ref-7] Compton WM, Dawson DA, Goldstein RB, Grant BF (2013). Crosswalk between DSM-IV dependence and DSM-5 substance use disorders for opioids, cannabis, cocaine and alcohol. Drug and Alcohol Dependence.

[ref-8] Compton WM, Volkow ND (2006). Major increases in opioid analgesic abuse in the United States: concerns and strategies. Drug and Alcohol Dependence.

[ref-9] Dart RC, Surratt HL, Cicero TJ, Parrino MW, Severtson SG, Bucher-Bartelson B, Green JL (2015). Trends in opioid analgesic abuse and mortality in the United States. New England Journal of Medicine.

[ref-10] DeAndrea DC, Troost JP, Anthony JC (2013). Toward primary prevention of extra-medical OxyContin^®^ use among young people. Preventive Medicine.

[ref-11] Grant BF, Harford TC, Dawson DA, Chou PS, Pickering RP (1995). The alcohol use disorder and associated disabilities interview schedule (AUDADIS): reliability of alcohol and drug modules in a general population sample. Drug and Alcohol Dependence.

[ref-12] Kanouse AB, Compton P (2015). The epidemic of prescription opioid abuse, the subsequent rising prevalence of heroin use, and the federal response. Journal of Pain Palliative Care Pharmacotherapy.

[ref-13] King NB, Fraser V, Boikos C, Richardson R, Harper S (2014). Determinants of increased opioid- related mortality in the United States and Canada, 1990–2013: a systematic review. American Journal of Public Health.

[ref-14] Lopez-Quintero C, Roth KB, Eaton WW, Wu LT, Cottler LB, Bruce M, Anthony JC (2015). Mortality among heroin users and users of other internationally regulated drugs: a 27-year follow-up of users in the Epidemiologic Catchment Area Program household samples. Drug and Alcohol Dependence.

[ref-15] Manchikanti L, Helm S, Fellows B, Janata JW, Pampati V, Grider JS, Boswell MV (2012). Opioid epidemic in the United States. Pain Physician.

[ref-16] Martins SS, Ghandour LA, Chilcoat HD (2007). Profile of dependence symptoms among extramedical opioid analgesic users. Addictive Behaviors.

[ref-17] Maxwell JC (2015). The pain reliever and heroin epidemic in the United States: shifting winds in the perfect storm. Journal of Addictive Diseases.

[ref-18] Meier EA, Troost JP, Anthony JC (2012). Extramedical use of prescription pain relievers by youth aged 12 to 21 years in the United States: national estimates by age and by year. Archives of Pediatrics and Adolescent Medicine.

[ref-19] Parker MA, Anthony JC (2014). Should anyone be riding to glory on the now-descending limb of the crack-cocaine epidemic curve in the United States?. Drug and Alcohol Dependence.

[ref-20] Seedall RB, Anthony JC (2015). Monitoring by parents and hypothesized male–female differences in evidence from a nationally representative cohort re-sampled from age 12 to 17 tears: an exploratory study using a “mutoscope approach”. Prevention Science.

[ref-21] StataCorp (2013). Stata statistical software: release 13.

[ref-22] United States Department of Health and Human Services (2006). The NSDUH Report—Nonmedical Users of Pain Relievers: Characteristics of Recent Initiates.

[ref-23] United States Department of Health and Human Services (2013). Results from the 2013 national survey on drug use and health: detailed tables.

[ref-24] United States Department of Health and Human Services (2014). Results from the 2013 national survey on drug use and health: summary of national findings. NSDUH series H-48, HHS publication no. (SMA) 14-4863.

[ref-25] United States Department of Health and Human Services (2015). National survey on drug use and health: 2-year R-DAS (2002 to 2003, 2004 to 2005, 2006 to 2007, 2008 to 2009, 2010 to 2011, and 2012 to 2013).

[ref-26] United States Centers for Disease Control and Prevention (2014). Prescription drug overdose in the United States: fact sheet. http://www.cdc.gov/homeandrecreationalsafety/overdose/facts.html.

[ref-27] Volkow ND, McLellan TA, Cotto JH, Karithanom M, Weiss SRB (2011). Characteristics of opioid prescriptions in 2009. Journal of the American Medical Association.

[ref-28] Vsevolozhskaya OA, Anthony JC (2014). Confidence interval estimation in R-DAS. Drug and Alcohol Dependence.

