# Supplementary material for: Epidemiological evidence on extra-medical use of prescription pain relievers: transitions from newly incident use to dependence among 12–21 year olds in the United States using meta-analysis, 2002–13
Source: PeerJ. 2015 Oct 20;3:e1340. doi: 10.7717/peerj.1340 (PMC4662579; doi:10.7717/peerj.1340)
Supplement: Supplemental Information 1 [file peerj-03-1340-s001.docx]

Newly incident users of extra-medical prescription pain relievers can be identified by the variable RECANL_B from the 2-year Restricted-use Data Analysis System (R-DAS). Year pairs from 2002 to 2003, 2004 to 2005, 2006 to 2007, 2008 to 2009, 2010 to 2011, and 2012 to 2013 are obtained via the year pair indicator (YRPRIND). Outlined in the 2011 NSDUH Summary of National Findings for 2002-10, the control totals are from analysis weights derived from the 2000 census data. For NSDUH weights after 2010, the 2010 census was used. We, therefore, are cautious in our comparisons between prescription pain reliever use estimates across these survey years (United States 2012).

Those who were eligible for past year initiation of pain reliever use can be identified using the R-DAS variable ELGANL_B. Those 'at risk' to become newly incident users were evaluated on the basis of the lifetime history of using prescription pain relievers extra-medically during an interval prior to the 12 months preceding the date of survey assessment. 'Ever users' who had a month and year of first use that pre-dated the interval were excluded from this 'at risk' population. Those eligible to become newly incident extra-medical prescription pain reliever users are designated by the RDAS variable ELGANL_B, coded with a value of 1.

RECANL_B and ELGANL_B pertain to 12 months before the assessment date and allow differentiation of newly incident users who started (or were eligible to start) using during that time interval, versus those who were 'at risk' but did not start using. For both RECANL_B and ELGANL_B, the NSDUH asks, “Have you ever, even once, used any type of prescription pain reliever that was not prescribed for you or that you took only for the experience or feeling it caused?”

All NSDUH questions about the use of pain relievers are not about "over-the-counter" pain relievers that can be obtained without a doctor's prescription. When asking about such use, the survey method involves presentation of a supplementary ‘Card A’ image to show pictures and names of various prescription pain relievers. Specific prescription pain relievers listed in the NSDUH questions include: Darvocet, Darvon, Tylenol with codeine, Percocet, Percodan, Tylox, Vicodin, Lortab, Lorcet, Codeine, Demerol, Dilaudid, Fioricet, Fiorinal, Hydrocodone, Methodone. Morphine, Oxycontin, Phenaphen, Propoxyphene, SK65, Stadol, Talacen, Talwin, Talwin-NX, Tramadol, and Ultram.

Dependence upon these compounds (DEPNDANL) was evaluated when there was extra-medical use of a prescription pain reliever in the 12 months prior to the date of assessment. The diagnostic criteria are those of the *Diagnostic and Statistical Manual for Mental Disorders, Fourth Edition* (DSM-IV; American Psychiatric Association, 1994). A user qualifies as a case of opioid dependence after evaluation in relation to standard DSM-IV criteria, including the withdrawal criterion, for opioid dependence: three or more of these seven criteria must be fulfilled as in the DSM-IV specifications. The withdrawal question was asked when opioid-specific withdrawal manifestations had lasted for longer than a day after cutting back or stopping use. DEPNDANL is a binary variable with only “Yes” or “No/Unknown (Otherwise)” as the alternative value labels.

Prevalence of recently active EMPPR use can be derived from the RDAS recoded variable IRANLRC. The original variable ANALREC asks, “How long has it been since you last used any prescription pain reliever that was not prescribed for you or that you took only for the experience or feeling it caused?” Answers vary between “Within the past 30 days,” “More than 30 days ago but within the past 12 mos,” “More than 12 months ago,” or “Never used pain relievers.” Combining the first two answer categories allows us to estimate prevalence of being a recently active user (within 12 months prior to assessment). Numerators for prevalence include those who used within the past 12 months.

Beginning with the 2002 NSDUH, missing values for the sex question (QD01) were not allowed so no imputation was required. This variable has the prefix ‘IR’, which stands for ‘Imputation Revised’, only for the sake of consistency with data sets from earlier surveys (pre-2002). The sex variable IRSEX has two values, “Male” and “Female”.

The 2-year RDAS file allows users to indicate age with the final edited age variable (AGE). After the respondent has entered his/her birthdate in the first part of the questionnaire, he/she has multiple opportunities to change his/her age in response to consistency checks throughout the questionnaire. The final age variable is determined using multiple survey questions. Age pairs were of interest in this analysis (e.g., 12-13; 14-15; 16-17; 18-19; 20-21) to match the paired nature of the years and ensure there would be no confidentiality issues due to small sample sizes. As noted in the text, these age values pertain to the participant's age on the date of assessment.

Note: NSDUH raw data files can be accessed via the following portal:

<http://www.icpsr.umich.edu/icpsrweb/NAHDAP/series/64>

As explained in our response to reviewers and correspondence, the use of the R-DAS portal to produce this study’s contingency tables is mid-stream between the ICPSR contract and a new contract vendor, as explained at this URL (last accessed 23 September 2015):

<http://www.icpsr.umich.edu/content/SAMHDA/index.html>

Nonetheless, ICPSR continues to distribute NSDUH public-use files that can be used by others to re-construct this study’s estimates, and the authors agree to provide Stata program code software as required to produce public use dataset versions of the R-DAS constructed variables used for this research report until the R-DAS portal has been re-opened by the new vendor in late 2015 or before mid-2016 (based on SAMHSA projections for that timetable). More information about the NSDUH public use datasets, and how to download them, can be found at this URL (last accessed 23 September 2015):

<http://www.icpsr.umich.edu/icpsrweb/NAHDAP/>.

**REFERENCES**

American Psychiatric Association (1994) DSM-IV: Diagnostic and Statistical Manual of Mental

Disorders (4th edition). Washington, DC: American Psychiatric Association

United States (2012) Results from the 2011 National Survey on Drug Use and Health: Summary

of National Findings. NSDUH Series H-44, HHS Publication No. (SMA) 12-4713. Rockville, MD: Substance Abuse and Mental Health Services Administration.

United States (2015) National Survey on Drug Use and Health: 2-Year R-DAS (2002 to 2003,

2004 to 2005, 2006 to 2007, 2008 to 2009, 2010 to 2011, and 2012 to 2013). Department of Health and Human Services. ICPSR34482-v3. Ann Arbor, MI: Inter-university Consortium for Political and Social Research [distributor]. http://doi.org/10.3886/ICPSR34482.v3
