# Supplementary material for: Epidemiological evidence on extra-medical use of prescription pain relievers: transitions from newly incident use to dependence among 12–21 year olds in the United States using meta-analysis, 2002–13
Source: PeerJ. 2015 Oct 20;3:e1340. doi: 10.7717/peerj.1340 (PMC4662579; doi:10.7717/peerj.1340)
Supplement: Supplemental Information 2 [file peerj-03-1340-s002.docx]

Drug researchers growing up during the interval from 1960 through 1990 had to deal with many shifts in the meaning of basic terms such as ‘drug’ and ‘drug misuse,’ as well as the more stigma-laden ‘drug abuse’ and ‘drug addiction’ terms. As time passed, the meanings of these terms became more and more ambiguous. In consequence, what philosopher John Locke described as the ‘signification’ of words became a problem. Ambiguity prevailed.

These ambiguities can be seen clearly in a simple comparison. First, we present what the Canadian ‘Le Dain’ Commission said in 1972 about ‘drug misuse’ and ‘drug abuse’ and we can compare that statement with what a recent US Food and Drug Administration (FDA) official said about these concepts. First, this is the excerpt from the Commission report:

We do not find the notion of drug "abuse" (or "misuse" for that matter) very helpful. In some cases it seems to be equated with the use of any drug which has a potential for producing dependence, physical or psychological. If it is equated with the drug use that actually produces dependence, then it is equated with only one potential aspect of harm. Certain kinds of drug use may produce harm quite apart from dependence, and in some cases, any use of a particular drug may involve the risk of harm. (<http://druglibrary.eu/library/reports/ledain/ldc6a-2.htm)>

In contrast, Dr. Klein’s more recent FDA view was as follows:

When a person takes a legal prescription medication for a purpose other than the reason it was prescribed, or when that person takes a drug not prescribed to him or her, that is misuse of a drug. Misuse can include taking a drug in a manner or at a dose that was not recommended by a health care professional. This can happen when the person hopes to get a bigger or faster therapeutic response from medications such as sleeping or weight loss pills. It can also happen when the person wants to “get high,” which is an example of prescription drug abuse. (<http://www.fda.gov/downloads/ForConsumers/ConsumerUpdates/UCM220434.pdf>)

By the late 1980s, the diversity of definitions and the loss of word signification for ‘drug misuse’ and ‘drug abuse’ prompted our research group to abandon these terms in our scientific reports. Stigma-laden terms such as ‘drug abuse’ and ‘drug addiction’ also became problems rather than solutions in scientific communication. For these reasons, we advocated a substitution with a more or less clear distinction between ‘medical’ and ‘extra-medical’ use of a drug.

Medical use of the drug was stipulated as use within the boundaries of a clinician-intended prescription or the equivalent as applied to over-the-counter non-prescription compounds.

Extra-medical use of the drug (as conveyed by the adjective stem ‘extra’) was stipulated as use ‘outside’ the boundaries of a clinician-intended prescription or the equivalent as applied to OTC non-prescription compounds.

After deciding to create a new term, we framed an operational specification for ‘extra-medical use’ as follows:

We are interested in the extra-medical use of these prescription-type drugs.

Extra-medical use is any use on your own; that is, either:

**One**, without a doctor's prescription, or

**Two**, in greater amounts than prescribed, or

**Three**, more often than prescribed, or

**Four**, for any reasons other than a doctor said you should take them-- such as for kicks, to get high, to feel good, or curiosity about the pill's effect.

Additional details about the ‘extra-medical drug use’ construct are provided in Sidebar 1 of this paper.
