# Supplementary figures and images for: Epidemiological evidence on extra-medical use of prescription pain relievers: transitions from newly incident use to dependence among 12–21 year olds in the United States using meta-analysis, 2002–13
Source: PeerJ. 2015 Oct 20;3:e1340. doi: 10.7717/peerj.1340 (PMC4662579; doi:10.7717/peerj.1340)

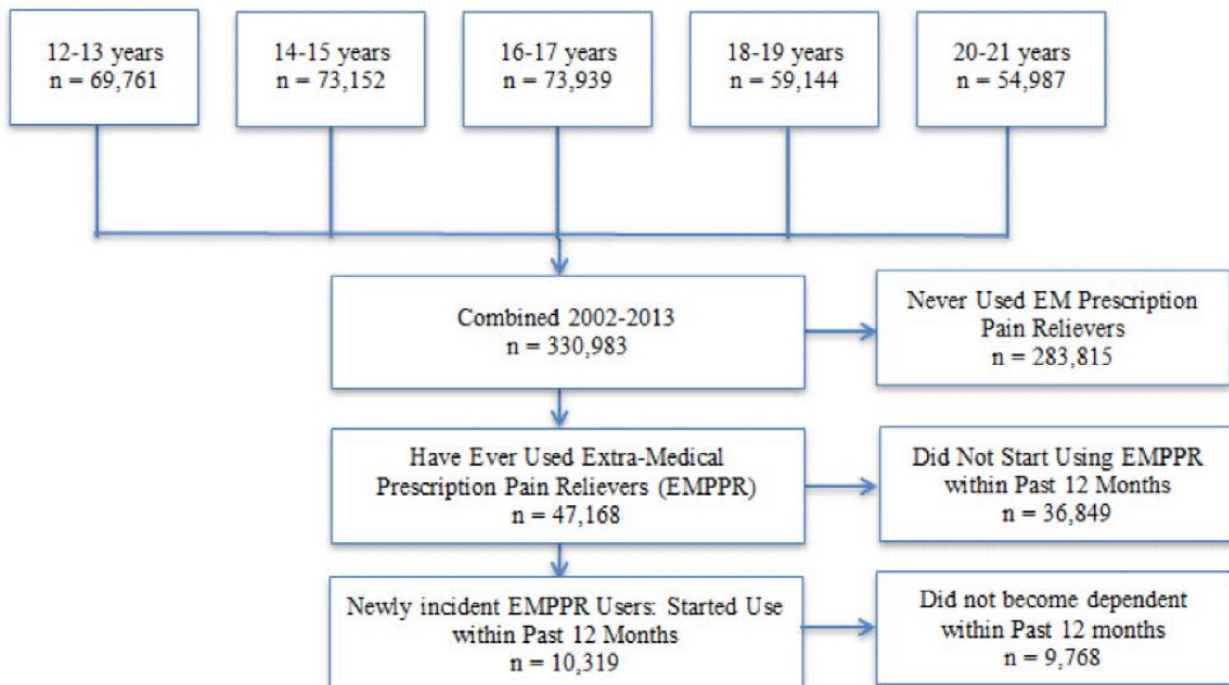

Supplement: Figure S1 — Data from the Survey Documentation and Analysis System of the National Surveys on Drug Use and Heath, United States 2002–2013. The NSDUH Restricted-use Data Analysis System (R-DAS) provides only weighted population estimates, but approximate unweighted sample sizes can be derived from NSDUH public use datasets archived with the Inter-university Consortium for Political and Social Research, as shown in this flow diagram. [file peerj-03-1340-s003.pdf]
