# Supplementary material for: Epidemiological evidence on extra-medical use of prescription pain relievers: transitions from newly incident use to dependence among 12–21 year olds in the United States using meta-analysis, 2002–13
Source: PeerJ. 2015 Oct 20;3:e1340. doi: 10.7717/peerj.1340 (PMC4662579; doi:10.7717/peerj.1340)
Supplement: Supplemental Information 4 [file peerj-03-1340-s004.docx]

In a meta-analysis of estimates from multiple years, the I-squared statistic serves as a measure of heterogeneity of these year-by-year estimates. When I-squared is small (and its p-value is large), there is less year-by-year variation and a fixed effects confidence interval can be justified. When I-squared is quite large (and its p-value is small), there is more year-by-year variation, and a 'random effects' confidence interval can be used to take the heterogeneity of estimates into account. The default 'fixed effects' estimator was specified, but the I-squared statistic has been used as a diagnostic tool for potential year-year variation.

In advance, we specified decision rules for the I-squared statistic as follows: (1) When the p-value for I-squared <0.05, the meta-analysis is re-specified so that the 'random effects' summary estimator is reported and used in subsequent analyses; (2) When the I-squared p-value is larger than 0.05 but less than 0.15, results based on both 'fixed effects' and 'random effects' summary estimators are presented; (3) Otherwise, the fixed effects summary estimate is presented, with considerable confidence that the between-year variability of estimates is acceptably small for the fixed effects approach. The 'metan' command was performed on the logit scale and transformed back to a proportion as illustrated by Vsevolozhskaya & Anthony (2014).

**REFERENCES**

StataCorp (2013) Stata Statistical Software: Release 13. College Station, TX: StataCorp LP

United States (2014) National Survey on Drug Use and Health: 2-Year R-DAS (2002 to 2003,

2004 to 2005, 2006 to 2007, 2008 to 2009, 2010 to 2011, and 2012 to 2013). Department of Health and Human Services. ICPSR34482-v3. Ann Arbor, MI: Inter-university Consortium for Political and Social Research [distributor]. http://doi.org/10.3886/ICPSR34482.v3

Vsevolozhskaya OA, Anthony JC (2014) Confidence interval estimation in R-DAS.

Drug Alcohol Depend. doi:10.1016/j.drugalcdep.2014.07.017
