# Supplementary material for: Epidemiological evidence on extra-medical use of prescription pain relievers: transitions from newly incident use to dependence among 12–21 year olds in the United States using meta-analysis, 2002–13
Source: PeerJ. 2015 Oct 20;3:e1340. doi: 10.7717/peerj.1340 (PMC4662579; doi:10.7717/peerj.1340)
Supplement: Figure S2 — The estimated risk of becoming a newly incident extra-medical user of prescription pain reliever drugs (A, leftmost columns) and the estimated risk of making a rapid transition and becoming an opioid dependence case no longer than 12 months after onset of starting to use these opioids extra-medically (B, rightmost columns). Risk estimates & 95% confidence intervals (per 100). Data from Restricted-use Data Analysis System subsamples of the National Surveys on Drug Use and Heath, United States 2002–2013. [file peerj-03-1340-s005.pdf]

Note 1: Understandably, the random effects meta-analysis estimating approach tends to yield less precise confidence intervals when replication samples produce heterogeneous estimates. There were some relatively minor but potentially important survey methods differences across the years (e.g., transition to full ACASI assessments in 2004-5; variations in US Census tables used for post-stratification adjustment factors). For this reason, we specified the random effects estimating approach, but substituted the fixed effects estimating approach when the heterogeneity test statistic's p-values were appreciably larger than the 0.05 level. When the heterogeneity test statistic's p-value was just slightly greater than 0.05, we have provided two forest plots, one of which is based on (and labeled as) the random effects plot and the other of which is based on (and labeled as) the fixed effects plot. In this fashion, readers have full information and can draw their conclusions based on whichever meta-analysis summary estimate and 95% CI might be preferred by them.

Note 2: Starting with 12-13 year olds, it seems that estimated risk of becoming a user might have declined, whereas estimated risk of transitioning from extra-medical use to dependence might have increased. However, please note the generally wide and overlapping confidence intervals, plus the fact that in the rightmost plots the estimated transitions to dependence generally had small heterogeneity test statistics (and large p-values), indicating that no inference should be drawn about any secular trend toward increasing risk of dependence upon newly incident users. In addition, given the relative imprecision and overlap of confidence intervals in the leftmost plots, plus the methods differences just noted, we would not wish to speculate about a secular trend in the direction of reduced incidence of use, based on this evidence. Some readers might enjoy speculating about these over-time 'tendencies, ' but we judge the estimates to be within the bounds of what might be expected under the generally arduous field conditions of large sample national surveys of this type. We also are mindful of the variations in the R-DAS post-stratification adjustment factors over time, which might well explain any observed heterogeneity. The R-DAS documentation and estimates do not make it possible for us to stabilize the post-stratification adjustment approaches and to rule out this source of observed heterogeneity in the estimates of becoming a newly incident extra-medical use of these compounds.

a. 12-13 year olds, becoming a user: random effects

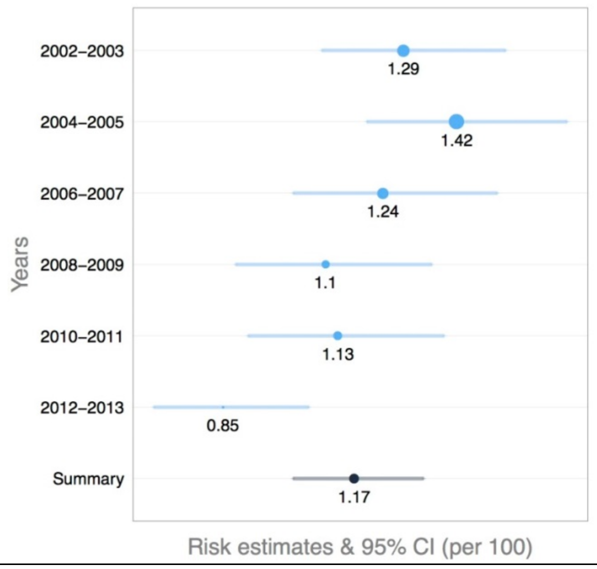

b. 12-13 year olds, transitioning from use to dependence: fixed effects

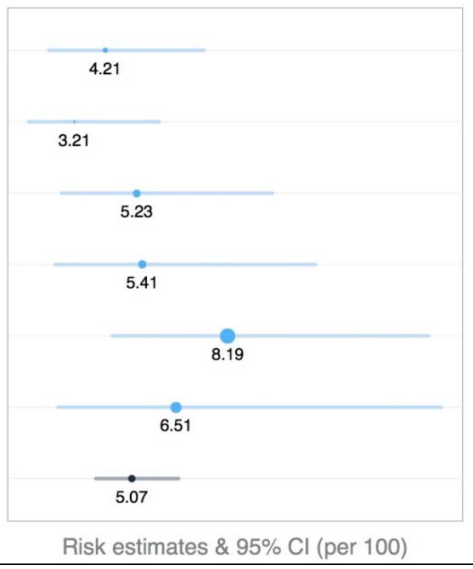

a. 14-15 year olds, becoming a user: random effects

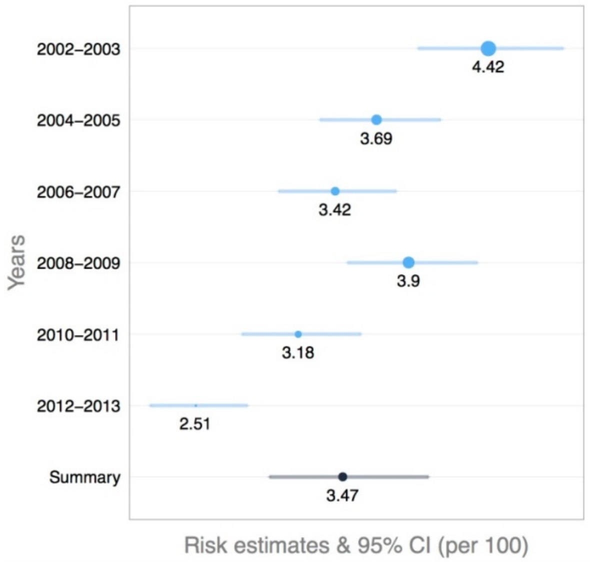

b. 14-15 year olds, transitioning from use to dependence: fixed effects

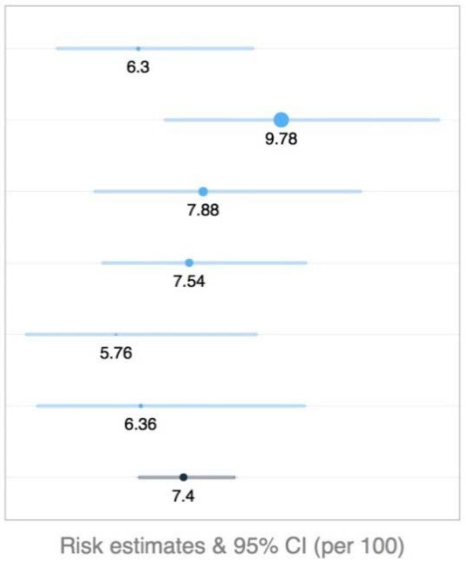

a. 16-17year olds, becoming a user: random effects

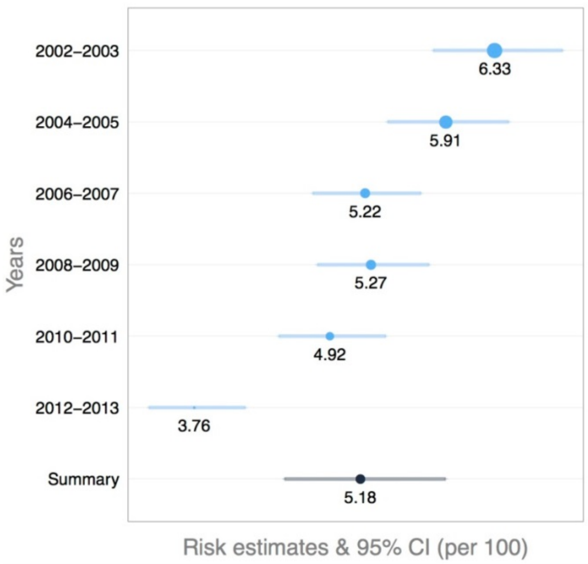

b. 16-17 year olds, transitioning from use to dependence: fixed effects

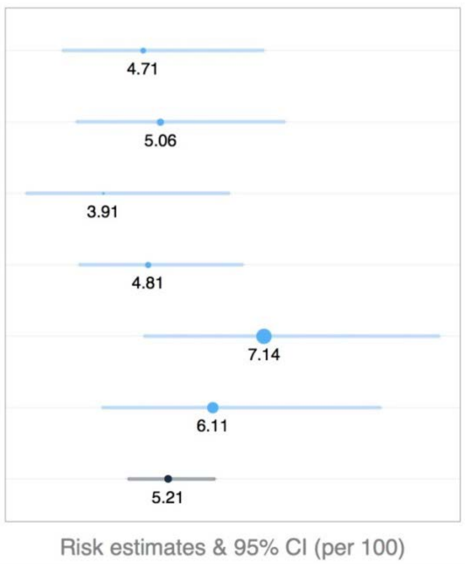

a. 18-19 year olds, becoming a user: random effects

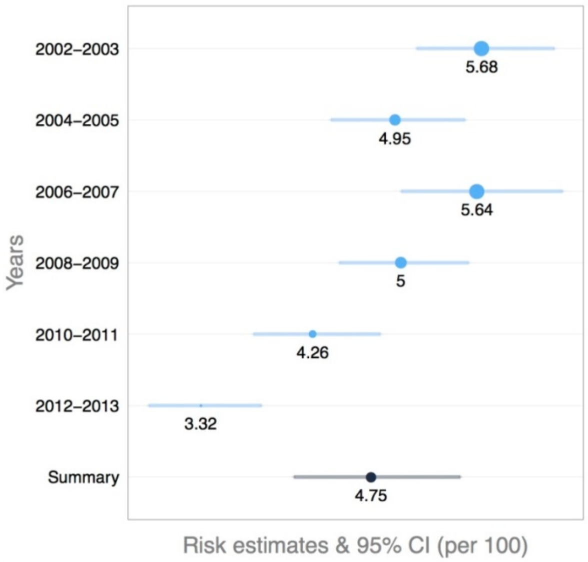

b. 18-19 year olds, transitioning from use to dependence: fixed effects

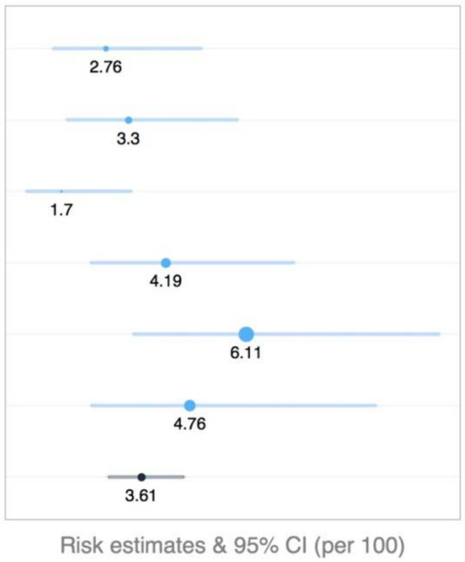

**a1. (large heterogeneity test statistic with p<0.05; therefore no fixed effects approach here)**	**b1. 18-19 year olds, transitioning from use to dependence: random effects**
	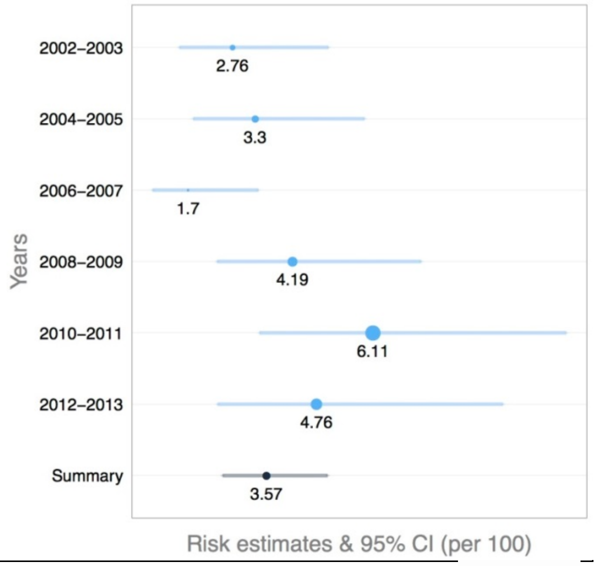

**a. 20-21 year olds, becoming a user: random effects**	**b. 20-21 year olds, transitioning from use to dependence: random effects**
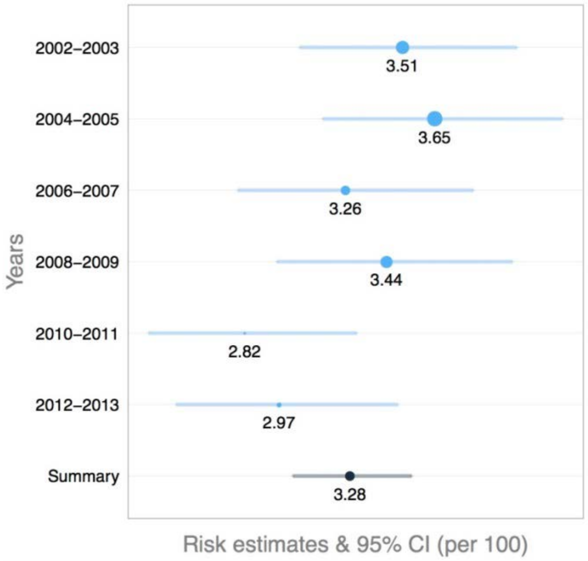	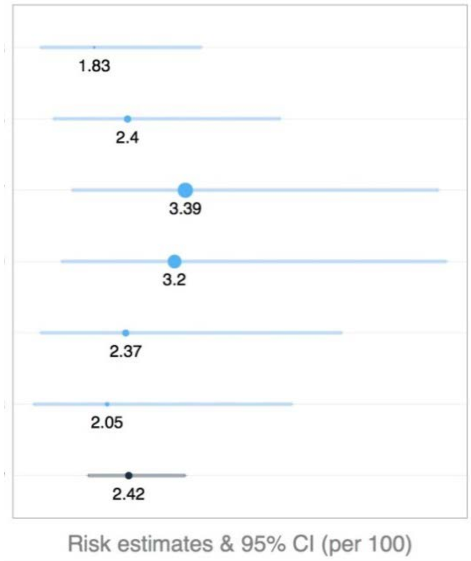

a1. 20-21 year olds, becoming a user: fixed effects	b1. (small heterogeneity test statistic with p>0.10; therefore no random effects approach here)
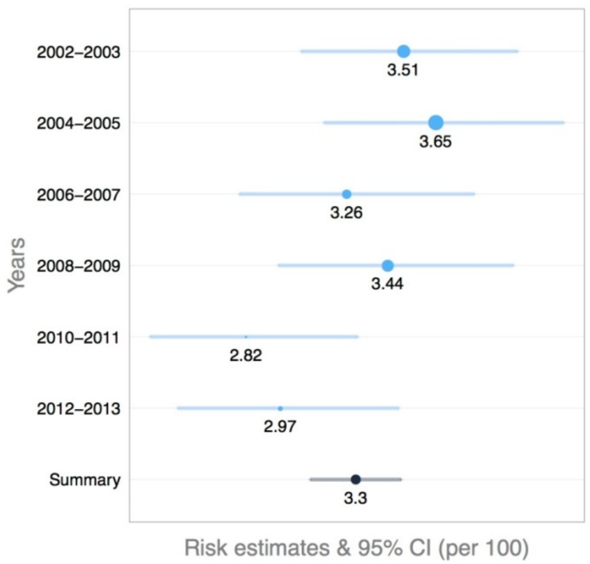	
