# Supplementary material for: Epidemiological evidence on extra-medical use of prescription pain relievers: transitions from newly incident use to dependence among 12–21 year olds in the United States using meta-analysis, 2002–13
Source: PeerJ. 2015 Oct 20;3:e1340. doi: 10.7717/peerj.1340 (PMC4662579; doi:10.7717/peerj.1340)
Supplement: Table S1 — Age- and time-specific incidence estimates (A), 95% confidence intervals (B), and age-specific meta-analysis summary estimates. Data from Restricted-use Data Analysis System subsamples of the National Surveys on Drug Use and Heath, United States 2002–2013. [file peerj-03-1340-s006.docx]

Table 1, Panel A: Estimated prevalence of being a user in the year prior to assessment (per 100)

| Year pair | 12-13 y | 14-15 y | 16-17 y | 18-19 y | 20-21 y |
| --- | --- | --- | --- | --- | --- |
| 2002-2003 | 3.2 | 7.6 | 12.1 | 13.3 | 12.5 |
| 2004-2005 | 3.4 | 6.7 | 11.3 | 13.8 | 13.5 |
| 2006-2007 | 3.0 | 6.8 | 10.7 | 13.7 | 13.2 |
| 2008-2009 | 2.9 | 6.4 | 9.9 | 12.3 | 12.6 |
| 2010-2011 | 2.9 | 5.8 | 9.5 | 10.7 | 10.9 |
| 2012-2013 | 2.3 | 4.8 | 7.6 | 9.3 | 9.9 |
|  | | | | | |
| **Table 2, Panel B: 95% Confidence intervals for estimates in Panel A (per 100)** | | | | | |
| Year pair | 12-13 y | 14-15 y | 16-17 y | 18-19 y | 20-21 y |
| 2002-2003 | 2.9, 3.5 | 7.0, 8.2 | 11.5, 12.8 | 12.6, 14.1 | 11.7, 13.3 |
| 2004-2005 | 3.0, 3.9 | 6.2, 7.2 | 10.6, 12.0 | 13.0, 14.6 | 12.6, 14.4 |
| 2006-2007 | 2.6, 3.4 | 6.3, 7.3 | 10.1, 11.3 | 12.9, 14.6 | 12.3, 14.1 |
| 2008-2009 | 2.5, 3.3 | 5.9, 6.9 | 9.3, 10.6 | 11.5, 13.1 | 11.8, 13.6 |
| 2010-2011 | 2.6, 3.3 | 5.3, 6.4 | 8.9, 10.1 | 10.0, 11.5 | 10.2, 11.8 |
| 2012-2013 | 2.0, 2.7 | 4.4, 5.3 | 7.1, 8.2 | 8.6, 10.1 | 9.1, 10.8 |
|  | | | | | |
| Meta-analysis summary estimates & 95% confidence intervals (per 100)^a^ | 3.7 (3.0, 3.3)^b^ | 6.8 (6.1, 7.6)^b^ | 10.8 (9.6, 12.1) ^b^ | 13.1 (12.1, 14.2) ^b^ | 13.0 (11.9, 14.1) ^b^ |

^a^ Supplement S4 provides additional information about I-squared.

^b^ I-squared statistic (p <0.05); these are the 'random effects' 95% confidence intervals.

^c^ I-squared statistic (0.05 > p > 0.15); the 'random effects' 95% confidence intervals = (3.0, 3.6).
