# Supplementary material for: Epidemiological evidence on extra-medical use of prescription pain relievers: transitions from newly incident use to dependence among 12–21 year olds in the United States using meta-analysis, 2002–13
Source: PeerJ. 2015 Oct 20;3:e1340. doi: 10.7717/peerj.1340 (PMC4662579; doi:10.7717/peerj.1340)
Supplement: Table S2 — Data from Restricted-use Data Analysis System subsamples of the National Surveys on Drug Use and Heath, United States 2002–2013. Offer Letter *Estimate derived via division of the Prevalence Estimate by the Incidence Estimate. [file peerj-03-1340-s007.docx]

| Estimate | 12-13 y | 14-15 y | 16-17 y | 18-19 y | 20-21 y |
| --- | --- | --- | --- | --- | --- |
| Incidence (per 100) | 1.2 | 3.5 | 5.2 | 4.8 | 3.3 |
| Prevalence (per 100) | 3.7 | 6.8 | 10.8 | 13.1 | 13.0 |
|  |  |  |  |  |  |
| Mean Duration* | 3.1 years | 2.0 years | 2.1 years | 2.8 years | 3.9 years |
